# Pharmacological Treatment Options for Coronavirus Disease-19 in Renal Patients

**DOI:** 10.1155/2021/4078713

**Published:** 2021-11-30

**Authors:** Jonny, Laurencia Violetta, Arief Sjamsulaksan Kartasasmita, Rully Marsis Amirullah Roesli, Coriejati Rita

**Affiliations:** ^1^Nephrology Division, Department of Internal Medicine, Gatot Soebroto Indonesia Army Central Hospital, Jakarta, Indonesia; ^2^Faculty of Medicine, Universitas Padjajaran, Bandung, West Java, Indonesia

## Abstract

Patients with chronic kidney disease (CKD), including dialysis and transplant patients, are at greater risk of contracting SARS-CoV-2 due to kidney dysfunction and preexisting comorbidities. To date, a specific guideline on managing these high-risk patients infected with COVID-19 has not been established. As the current management of COVID-19 comprises mainly experimental drugs, the authors aim to provide information on dosing adjustments at different stages of kidney dysfunction and notable renal side effects. We performed a nonsystematical review of currently available COVID-19 drugs exploring several different clinical trial databases and search browsers. Several antivirals and monoclonal antibodies used in COVID-19 treatment require dosage adjustments in kidney dysfunction. In a global pandemic setting, nephrologists need to consider the appropriate dosage according to the renal function and closely monitor the side effects of different drug combinations to obtain the optimum therapeutic effect while avoiding further renal damage. Further studies are required to determine the safety and efficacy of these drugs in renal patients.

## 1. Introduction

With severe acute respiratory syndrome coronavirus 2 (SARS-CoV-2) ability to bind with the angiotensin-converting enzyme 2 receptors, the kidney becomes one of the potential target organs. The presence of acute kidney injury (AKI) in COVID-19 is associated with disease severity and poor prognosis [1]. COVID-19-associated AKI is caused by direct viral damage of renal tubules and activation of proinflammatory cytokines leading to endothelial dysfunction and renal hypoperfusion [2]. Renal involvement in COVID-19 may occur in 0.5–80.3% of patients, in which a higher percentage of AKI occurs in critically ill patients [3]. Meanwhile, chronic kidney disease (CKD) can be commonly found in the population, contributing to immune dysfunction and immunodeficiency, increasing susceptibility to infection. Furthermore, comorbidities such as old age, diabetes, and hypertension are the known risk factors of COVID-19-related mortality [4]. Transplant patients are also vulnerable to acquiring severe infection due to chronic use of immunosuppressants [5].

Nopsopon et al. [6] reported a higher prevalence of COVID-19 in end-stage kidney disease (ESKD) patients than the average prevalence globally (3.1% vs. 0.1%). This population may also be at higher risk of dying from COVID-19. CKD patients had a three-fold risk of acquiring severe COVID-19 [7], where preexisting CKD can be found in 57.1% of COVID-19 patients in the intensive care unit (ICU) [3]. The mortality of patients with CKD stages 3–5, hemodialysis (HD), and transplant infected by COVID-19 were considerably higher than those without kidney disease (28.4%, 16.2%, and 11.1% vs. 4%, respectively) [8]. Similarly, the presence of COVID-19 significantly increased the mortality rate of patients on dialysis (20.2% vs. 0.2%) and transplant recipients (21.2% vs. 1.2%) compared to non-COVID patients [9].

Patients receiving in-center dialysis are at an additional two-fold risk of acquiring SARS-CoV-2 infection, with three to four times more significant risk of COVID-19-related hospitalization than those receiving home dialysis, contributed by increased contact with other patients and medical workers [10, 11]. The Centers for Disease Control and Prevention (CDC) and the American Society of Nephrology (ASN) have issued guidance to mitigate the risk of COVID-19 transmission in dialysis facilities, including strict surveillance protocols for patients and medical personnel before entering the facility, limiting outpatient visits to only medical emergencies, instructing the use of proper personal protective equipment and social distancing policies, and routine disinfection of the facilities [12].

To date, no clear guidelines exist for the management of COVID-19 in renal patients. The BNT162b2 mRNA and mRNA-1273 vaccines demonstrated promising protective effects and safe use in dialysis and transplant patients [13]. So far, remdesivir is the only drug approved by the Food and Drug Association (FDA), while several monoclonal antibodies and immunomodulators have obtained the emergency use authorization (EUA) for COVID-19 [14]. Due to disease novelty, the current management strategy of COVID-19 has been mainly preventive and supportive with the practical use of various medications. Some of these medications or their metabolites are substantially eliminated in the kidney, which may increase the risk of toxicity in patients with kidney dysfunction. The presence of underlying renal disease creates a challenge for nephrologists to utilize the current therapeutic options and prescribe appropriately to avoid further injury to the kidney.  Table 1 summarizes the drugs used in COVID-19 management based on the available evidences and recommendations, with the proposed dosing adjustment in different stages of kidney dysfunction and potential renal adverse events.

## 2. Systemic Corticosteroids

Corticosteroids decrease proinflammatory cytokines and suppress the “cytokine storm” involved in rapid clinical deterioration and death [15]. The RECOVERY trial preliminary findings reported a significant mortality reduction with dexamethasone, particularly in severe and critical diseases [16]. Since then, several RCTs evaluating corticosteroids in COVID-19 have emerged, supporting this finding, with a 28-day mortality reduction of 6.7% and 8.7% in severe and critically ill patients, respectively [17]. A strong recommendation was given to corticosteroids for severe and critically ill COVID-19, but not for those in the early phases of infection or nonsevere COVID-19, ideally starting seven days since symptom onset [18]. No dose adjustments are necessary for renal patients, although routine monitoring is advised. They are also not significantly affected by renal replacement therapy. Thus, regular doses may be used for dialysis patients [19].

### 2.1. Antivirals

#### 2.1.1. Remdesivir

Remdesivir inhibits viral RNA-dependent RNA polymerase (RdRp), leading to premature RNA termination and halting transcription [20]. Several trials reported that remdesivir significantly reduced recovery time but not mortality or length of hospital stay [21, 22]. Nevertheless, the FDA has approved its use as COVID-19 treatment for hospitalized adult and pediatric patients requiring minimal supplemental oxygen [14]. All patients must have their estimated glomerular filtration rate (eGFR) to be determined before treatment, and patients with eGFR <30 ml/min are not recommended to receive remdesivir. Potential renal toxicity stems from 50% of its active metabolite (GS-44I524) being excreted in the urine. As an RdRp inhibitor, the risk of mitochondrial injury may occur, although animal studies showed that injury only occurs at a considerably higher dose (5–20 mg/kg/day) and after prolonged exposure [14]. Furthermore, each 100 mg vial contains up to 3–6 g of a drug vehicle, sulfobutylether-*β*-cyclodextrin (SBECD), renally excreted, and may accumulate with reduced renal function. SBECD is readily eliminated by dialysis, which maintains its accumulation within a generally safe limit [23]. Remdesivir at a dose of 2.5 mg/kg of patients' dry weight, up to 100 mg given 4 hours before dialysis, was well tolerated in HD patients [24].

#### 2.1.2. Lopinavir/Ritonavir

Lopinavir/ritonavir (LPN/r) is a protease inhibitor that interferes with normal viral replication and growth. LPN/r has been described to reduce viral load and acute respiratory distress syndrome [25]. However, early randomized clinical trial (RCT) in severe COVID-19 revealed no benefit in terms of safety and reducing mortality [26]. A similar finding was also described in the SOLIDARITY trial's interim results; nonetheless, the LPN/r group had a numerically lower mortality rate and shorter ICU stay [27]. Approximately 10% of LPN is renally excreted, and its plasma concentration is not altered by renal impairment or dialysis [14].

#### 2.1.3. Favipiravir

Favipiravir is another RdRp inhibitor currently under investigation for SARS-CoV-2 (NCT04425460 and NCT04336904). Favipiravir resulted in faster viral clearance and radiological improvement with fewer adverse events compared to LPN/r [28]. Several meta-analyses have demonstrated the similar benefit of favipiravir compared with standard care or other antivirals [29, 30]. No clear data are available regarding dose adjustment in impaired renal function. It is predominantly eliminated in the urine (>90%), thus not recommended to be used in patients with eGFR <30 ml/min. No recommendation is available on favipiravir use for dialysis patients. However, it is reported that its blood concentrations in HD patients were similar to those in patients with normal renal function, suggesting its potential use in treating dialysis patients [31].

#### 2.1.4. Darunavir/Cobicistat

Darunavir is a protease inhibitor that has been shown in silico to have high binding activity to SARS-CoV-2 protease. Cobicistat is added to prevent darunavir's inhibition by cytochrome P450 [32]. Darunavir/cobicistat (DRV/c) can be considered an alternative for COVID-19 under several clinical trials (NCT04425382 and NCT04252274). Like LPN/r, darunavir has almost negligible renal clearance and is highly protein-bound, thus not easily eliminated by dialysis [14].

#### 2.1.5. Danoprevir/Ritonavir

Danoprevir/ritonavir is a potent inhibitor of hepatitis C virus protease that is repurposed for COVID-19. The early trial revealed that danoprevir/ritonavir is safe and well-tolerated, reducing viral replication and improving clinical symptoms [33]. The efficacy of this combination for COVID-19 is still currently under investigation (NCT04345276 and NCT04291729). In addition, no data are yet available regarding dosage adjustments for renal impairment and their renal adverse effects.

#### 2.1.6. Ribavirin

Ribavirin is a guanosine analogue that disrupts RNA translation and viral replication and possesses antiviral activity against SARS-CoV-2 in vitro [34]. Ribavirin as an add-on to LPN/r and interferon beta-1b for mild-to-moderate COVID-19 resulted in a shorter duration of viral shedding and hospital stay than LPN/r alone [35]. However, no evidence is available regarding its benefit in reducing the mortality rate. The plasma concentration of ribavirin increases with a decline in creatinine clearance. Dosage adjustment for renal dysfunction is well-tolerated and delivers similar plasma ribavirin coverage to that observed among patients without renal impairment [14].

#### 2.1.7. Oseltamivir

Oseltamivir is a prodrug that inhibits viral neuraminidase and is initially administered empirically in the Wuhan outbreak. However, further studies did not find its usefulness against SARS-CoV-2 since it lacks neuraminidase [36]. Currently, oseltamivir is still under investigation, used with other antivirals or antibiotics (NCT04255017 and NCT04303299). Since 99% of its active metabolite, oseltamivir carboxylase, is excreted entirely through the kidney, doses should be lowered to avoid metabolite accumulation [14].

#### 2.1.8. Chloroquine and Hydroxychloroquine

Antimalarial agents, chloroquine (CQ) and hydroxychloroquine (HCQ), were among the first repurposed drugs in COVID-19 treatment. Their antiviral properties were related to the interference of SARS-CoV-2 binding to angiotensin-converting enzyme 2, replication and viral entry, as well as inhibition of cytokine release and CD8+ T cell activity [37]. Their efficacy for COVID-19 has been controversial. Early trials demonstrated that HCQ + azithromycin could reduce viral load significantly, resulting in significant absorption of pneumonia and reduced recovery time [38, 39]. However, results from larger RCTs (SOLIDARITY and RECOVERY trials) found little to no benefit of HCQ, alone or with azithromycin, in reducing short-term mortality or hospital stay in hospitalized COVID-19 patients [27, 40]. For mild-to-moderate COVID-19, HCQ also did not benefit from improving clinical status while causing the more frequent side effect of QT prolongation [41]. Therefore, the use of CQ or HCQ, with or without azithromycin, was not recommended for hospitalized and nonhospitalized COVID-19 patients. Renal clearance of HCQ is not associated with creatinine clearance; thus, dosage adjustment is not required. However, since the kidney substantially excretes them (40–50%), there is an increased risk of toxicity with impaired renal function [14]. For short-term use, no dosage adjustment is necessary for dialysis patients; however, these drugs should be administered after dialysis sessions to avoid drug loss [42].

### 2.2. Antibiotics

Azithromycin and doxycycline are commonly used antibiotics repurposed as COVID-19 treatment due to their potential antiviral and immunomodulatory properties. The addition of azithromycin to HCQ has a synergistic effect in reducing viral load [38]. However, it was not shown to improve outcomes in critically ill patients, with azithromycin addition being considered to cause more harm from severe QT prolongation [41]. Azithromycin efficacy for mild-to-moderate COVID-19 is still under investigation (NCT04381962 and NCT04338698). Given the cardiotoxicity risk, doxycycline could be a better alternative to be combined with HCQ. In a case series of COVID-19 patients with pulmonary comorbidities, doxycycline effectively improved clinical symptoms with no safety issues. Doxycycline combined with ivermectin was shown to reduce the mortality rate from 22.72% to 0% in severe COVID-19 [43]. However, the recent PRINCIPLE trial's interim results reported that neither antibiotics speed up recovery or hospitalization stay compared to standard care. Both drugs undergo minimal renal elimination and have not been shown to accumulate in renal impairment, with a small fraction of unchanged azithromycin and doxycycline appearing in urine. Standard doses of both antibiotics can be used in dialysis patients as they are known not to be removed by dialysis [44].

### 2.3. Monoclonal Antibody Agents

The FDA issued EUA for several anti-SARS-CoV-2 monoclonal antibodies as a treatment for outpatient mild-to-moderate COVID-19 patients who are at high risk for deterioration to severe disease or requiring hospitalization, including patients with CKD and those receiving immunosuppressive treatments [18].

#### 2.3.1. Bamlanivimab/Etesevimab

Bamlanivimab is a monoclonal antibody that binds to the spike protein of SARS-CoV-2 and prevents its entry into host cells. The BLAZE-1 study's interim results showed its benefit in lowering viral load and risk of hospitalization and death [45]. In November 2020, the FDA issued a EUA for bamlanivimab monotherapy. However, the EUA has been revoked due to increasing SARS-CoV-2 variants' resistance to bamlanivimab. The updated NIH COVID-19 treatment guideline instead recommended using a combination of bamlanivimab plus etesevimab to treat mild-to-moderate COVID-19 as the combination maintains its activity against the SARS-CoV-2 delta variant [18]. No dose adjustment is recommended in those with renal impairment [14].

#### 2.3.2. Casirivimab/Imdevimab

Casirivimab and imdevimab (REGEN-COV) are recombinant human monoclonal antibodies that work in synergy to inhibit SARS-CoV-2 binding from host cells. REGEN-COV also received EUA to treat nonhospitalized COVID-19 patients. A phase 3 RCT reported a 70% relative reduction in hospitalization or death among outpatient COVID-19 patients [18]. These drugs are not eliminated in urine, and dialysis is not expected to impact their elimination [14].

#### 2.3.3. Sotrovimab

Sotrovimab is a monoclonal antibody that binds to the spike of SARS-CoV-2 protein and has been granted EUA to treat outpatient mild-to-moderate COVID-19. The COMET-ICE trial reported the efficacy of sotrovimab in reducing the rate of hospitalization or death (80%) among the sotrovimab group compared to controls [18]. No dosage adjustment is required in renal impairment, since the drug is not eliminated intact in urine [14].

#### 2.3.4. Tocilizumab

Tocilizumab is an antiinterleukin (IL)-6 receptor monoclonal antibody that results in inflammation reduction. An initial pilot study showed significant improvement in ferritin, C-reactive protein, and D-dimer levels after tocilizumab, with early use associated with increased survival in severe COVID-19 [46]. However, the latest phase 3 COVACTA trial reported no clinical benefit of tocilizumab in terms of mortality rate or ventilator-free days compared to controls [47]. Tocilizumab is not renally excreted, and no dose adjustment is required in mild-to-moderate renal impairment, although its use has not been studied in severe impairment. No dose adjustments are required in dialysis since the considerable molecular weight of tocilizumab avoids clearance via dialysis [14].

#### 2.3.5. Baricitinib

Baricitinib is an inhibitor of Janus kinase that prevents immune activation and inflammation. As of November 2020, baricitinib, in combination with remdesivir, received EUA as COVID-19 treatment [18]. The ACTT-2 trial showed that baricitinib plus remdesivir resulted in a significantly faster recovery time, although no difference in short-term mortality was observed compared with remdesivir only [48]. The kidney excretes approximately 75% of this drug, and the risk of adverse reactions increases as renal function decreases. Dosage adjustment is required in those with eGFR <60 ml/min. Furthermore, approximately 75% of baricitinib is excreted by the kidney, which increases the risk of adverse reactions as renal function decreases. Baricitinib is not recommended for dialysis patients or those who develop AKI [14].

#### 2.3.6. Adalimumab

Adalimumab targets tumor necrosis factor (TNF)-*α* explicitly. Data from the SECURE-IBD [49] and COVID-19 Global Rheumatology Alliance [50] registries demonstrated that anti-TNF therapies in patients with inflammatory bowel disease and rheumatic disease infected with COVID-19 were associated with lower hospital admission, ventilator use, and death. Its effectiveness for COVID-19 in the community settings is currently under the preenrollment phase [51]. Adalimumab can be administered without dose adjustment and was shown to be safe and well-tolerated in CKD patients, including those undergoing dialysis, with no significant worsening of renal function after 24-month follow-up [52].

#### 2.3.7. Sarilumab

Sarilumab is another anti-IL-6 receptor monoclonal antibody that modulates IL-6 levels associated with severe COVID-19 [18]. Its efficacy is currently under investigation (NCT04315298). Early results showed early results of CRP level reduction, although no clinical benefit was observed. Whether early administration can prevent cytokine storms and subsequent ICU admission is under exploration in the SARICOR trial (NCT04357860). Sarilumab is not renally excreted; thus, dose adjustment is not required in mild-to-moderate renal dysfunction, but its use has not been studied in severe impairment [14]. Intravenous sarilumab has been recommended as an alternative to tocilizumab when it is not available [18].

## 3. Discussion and Conclusion

Renal involvement in COVID-19 presenting with hematuria and proteinuria is an independent predictor of in-hospital mortality [1]. CKD is a known comorbidity of severe COVID-19, while AKI is a common complication of severe COVID-19, both creating a challenge for physicians in treating COVID-19 in this population [2]. Two centers in Italy have reported their experience in managing COVID-19 within the nephrology ward [19, 53]. Both authors adopted a 2-phase pharmacological approach in treating COVID-19: (1) antivirals and azithromycin for the first phase of infection (within 7–10 days from symptom onset) and (2) immunosuppressive and immunomodulatory drugs during the second phase. Dialysis units should provide strict isolation measures since patients undergoing in-center dialysis are especially vulnerable to contracting COVID-19. It is also recommended that critically ill COVID-19 patients who developed AKI or had the indications to receive RRT should use continuous renal replacement therapy with the hemodiafiltration method, or else, use medium cutoff membranes in patients who undergo dialysis to increase the removal of inflammatory mediators [18, 19].

The International Society of Peritoneal Dialysis guideline stated that COVID-19 management of PD patients is similar to other patients. Mild-to-moderate patients can continue PD, while severe or critical cases should be temporarily transferred to automated PD or continuous renal replacement therapies or intensify ultrafiltration if the patient decides to remain on PD. In addition, telehealth should be used extensively to communicate with PD patients remotely [54]. Kidney donors should be screened for COVID-19, and those with symptoms or travel history to high-risk areas should delay kidney donation up to 14–28 days [55]. No standardized guideline is available regarding managing transplant patients infected with COVID-19 since using immunosuppressants in COVID-19 is conflicting. Overall, the decision on immunosuppression should be individualized based on the specific clinical scenario [56]. More information is needed to determine the optimal timing of discontinuation and reintroduction of immunosuppressants.

In the setting of a global pandemic, a drug repurposing strategy is necessary to find effective therapy, creating a challenge for nephrologists in managing renal patients infected with COVID-19 due to the potential renal side effects. Most of the drugs in this review are not renally excreted and may be applied in patients with renal dysfunction. However, several antivirals and monoclonal antibody agents, such as remdesivir, favipiravir, ribavirin, oseltamivir, baricitinib, and tofacitinib, are renally eliminated, which require dosage adjustment, and their use is generally contraindicated in eGFR <30 ml/min. The available data did not show an increased risk of renal side effects in patients with AKI, CKD, and those on dialysis receiving remdesivir [23, 57]. As RdRp inhibitors, remdesivir and favipiravir pose the risk of mitochondrial injury. In particular, favipiravir was reported to cause a reversible elevation of blood uric acid level [58]. While darunavir does show any effect on renal function, cobicistat is known to inhibit MATE1, a transporter responsible for creatinine excretion into urine, which may result in a reversible creatinine increase. However, no effect was observed on the actual eGFR [32].

Acute interstitial nephritis (AIN) is often associated with medications including lopinavir/ritonavir, azithromycin, and adalimumab, which warrants close monitoring of renal function timely discontinuation if AIN is suspected [59–61]. Potential nephrolithiasis may occur in patients receiving tocilizumab, although the incident was reported to be less than 2% in previous studies [14]. Previously commonly used antimalarial agents, HCQ and CQ, are substantially excreted by the kidney (40–50%), requiring close kidney function monitoring in renal patients. Furthermore, both drugs have been reported to induce renal phospholipidosis mimicking Fabry disease, although this is unlikely to occur with short-term use [42]. Overall, the short treatment duration (5–14 days) may suggest that these renal adverse events may be infrequent. Nonetheless, kidney diseases are characterized by impairment of the host immune system; therefore, renal function should be monitored carefully to avoid aggravating kidney damage when deciding to prescribe these medications, particularly those with immunosuppressant properties, based on the professional judgement that the benefits may outweigh the potential risks. Unfortunately, the efficacy and safety of these medications in renal patients are limited as patients with kidney dysfunction are often excluded from trials, which warrants the need to include this specific population in future COVID-19 drug trials.

## Figures and Tables

**Table 1:** Drugs used to manage COVID-19 (alone or in combination) with their proposed dosing adjustment in different stages of chronic kidney disease and potential renal adverse events.

Drug	COVID-19 status	Normal dose (eGFR ≥90)	Dose according to different stages of renal disease					Renal adverse events
			eGFR 60–89	eGFR 30–59	eGFR <30	Dialysis ^*∗*^	Transplant	
Systemic corticosteroids								
Dexamethasone	Phase III	6 mg/24 h po/iv (7–10 days) or days 1–5: 20 mg/24 h; days 6–10: 10 mg/24 h	Normal dose	Normal dose	Normal dose	Normal dose	Normal dose	Not reported
Hydrocortisone	Phases II-III	50 mg/8 h iv (7–10 days)	Normal dose	Normal dose	Normal dose	Normal dose	Normal dose	Not reported
Methyl-prednisolone	Phase III	8 mg/6 h iv (7–10 days)	Normal dose	Normal dose	Normal dose	Normal dose	16 mg followed by 8–16 mg/day	Not reported
Antivirals								
Remdesivir	Phase III	200 mg/24 h iv (day 1) and then 100 mg/24 h iv (up to 5–10 days)	Normal dose	Normal dose	Avoid use	HD: may be used (100–200 mg given 4 hours prior)	Based on eGFR	Mitochondrial injury in tubular epithelial cells
						PD: N/A		SBECD-associated renal toxicity

Darunavir/cobicistat	Phase III	800 mg/150 mg/24 h po (5–14 days)	Normal dose	Normal dose	Normal dose	Normal dose	Normal dose	Cobicistat-associated false creatinine elevation
Danoprevir/ritonavir	Phase IV	100 mg/100 mg/12 h po (10–14 days)	N/A	N/A	N/A	N/A	N/A	N/A
Ribavirin	Phase II	400 mg/12 h (14 days)	Normal dose	200 mg and 400 mg every other day)	200 mg/24 h	200 mg/24 h	Based on eGFR	Not reported
Oseltamivir	Phase IV	75 mg/12 h po (5 days)	Normal dose	75 mg/24 h or 30 mg/12 h po	eGFR 10–30: 30 mg/24 h po	HD: 30 mg after each session (3x/week)	Based on eGFR	Not reported
					eGFR<10 not on dialysis: avoid the use	PD: 30 mg single dose after or 30 mg weekly		

Hydroxychloroquine	Phases III-IV	Day 1: 400–600 mg/24 h po; days 2–5: 200 mg/12 h po	200 mg/12 h po	200 mg/12 h po	eGFR 15–30: 200 mg/24 h po	Normal dose (administer after dialysis)	Based on eGFR	Renal phospholipidosis
					eGFR <15: 200 mg po alternate days			
Chloroquine	Phase IV	Day 1: 600 mg and then 300 mg after 12 h po; days 2–5: 300 mg/12 h po or 500 mg/12 h po (5–10 days)	Normal dose	Normal dose	eGFR >10: normal dose	Normal dose (administer after dialysis)	Based on eGFR	Renal phospholipidosis
					eGFR <10: reduce dose by 50%			

Antibiotics								
Azithromycin	Phase IV	500 mg/24 h po (3–5 days) or 500 mg (day 1), 250 mg/24 h (days 2–5)	Normal dose	Normal dose	Normal dose	Normal dose	Normal dose	Potential drug-induced acute interstitial nephritis
Doxycycline	Phase III	100 mg/12 h po (5 days)	Normal dose	Normal dose	Normal dose	Normal dose	Normal dose	Not reported
Monoclonal antibody agents								
Bamlanivimab + etesevimab	Phase III	700 mg/1400 mg iv once	Normal dose	Normal dose	Normal dose	Normal dose	Normal dose	Not reported
Casirivimab + imdevimab	Phase III	600 mg/600 mg iv once	Normal dose	Normal dose	Normal dose	Normal dose	Normal dose	Not reported
Sotrovimab	Phase III	500 mg iv infusion once	Normal dose	Normal dose	Normal dose	Normal dose	Normal dose	Not reported
Tocilizumab	Phase III	8 mg/kg iv (max 800 mg)	Normal dose	Normal dose	Normal dose	Normal dose	Normal dose	Nephrolithiasis
Baricitinib	Phase III	4 mg/24 h po (14 days)	Normal dose	2 mg/24 h po	eGFR 15–30: 1 mg/24 h po; eGFR <15: avoid use	Avoid use	Avoid use	Acute kidney injury
Adalimumab	Phase IV	160 mg sc once	Normal dose	Normal dose	Normal dose	Normal dose	Normal dose	Granulomatous acute interstitial nephritis
Sarilumab	Phases II-III	200–400 mg iv once	Normal dose	Normal dose	Normal dose	Normal dose	Normal dose	Not reported

^*∗*^Dialysis includes hemodialysis and peritoneal dialysis. eGFR, estimated glomerular filtration rate; po, peroral; iv, intravenous; sc, subcutaneous.
